# Supplementary material for: Identification of Key Candidate Genes for Muscle Growth in Liaoning Black Pigs and Duroc Pigs via Longissimus Dorsi Muscle Transcriptome Analysis
Source: Curr Issues Mol Biol. 2025 Nov 5;47(11):917. doi: 10.3390/cimb47110917 (PMC12651075; doi:10.3390/cimb47110917)
Supplement: Supplementary file 1 [file cimb-47-00917-s001.zip › cimb-3924655-supplementary.pdf]

Sequencing Data Table 1

Sample	Raw Reads	Raw Bases	Clean Reads	Clean Bases	Error Rate	Q20	Q30	GC Content
CH1	40863870	6.12G	40160678	6.02G	0.03%	97.84%	94.13%	50.72%
CH2	40818096	6.12G	40117344	6.02G	0.03%	97.88%	94.23%	51.28%
CH3	42574662	6.38G	41718406	6.26G	0.03%	97.91%	94.27%	51.00%
CH4	44316398	6.64G	43729000	6.56G	0.03%	97.85%	94.05%	52.54%
CH5	47338284	7.1G	46658854	7G	0.03%	97.92%	94.28%	52.51%
CH6	46906452	7.03G	46354346	6.95G	0.03%	97.77%	93.89%	52.28%
HD1	44968386	6.74G	44239066	6.64G	0.03%	97.88%	94.15%	50.73%
HD2	44126990	6.61G	43430136	6.51G	0.03%	97.76%	93.83%	50.20%
HD3	42805394	6.42G	42230762	6.33G	0.03%	97.89%	94.17%	51.20%
HD4	41145384	6.17G	40540542	6.08G	0.02%	97.96%	94.34%	49.41%
HD5	41677596	6.25G	40996270	6.15G	0.03%	97.64%	93.62%	49.40%
HD6	42975672	6.44G	42195386	6.33G	0.03%	97.89%	94.23%	49.63%
